# Left Atrial Dysfunction in Patients with Atrial Fibrillation and Heart Failure

**DOI:** 10.1007/s11886-025-02324-6

**Published:** 2025-11-27

**Authors:** Cory R. Trankle, Ajay Pillai, Hussein Krayem, Keyur B. Shah, Kenneth A. Ellenbogen, W. Gregory Hundley

**Affiliations:** 1 Division of Cardiology, VCU Pauley Heart Center, Virginia Commonwealth University, Richmond, VA USA; 2 VCU Pauley Heart Center, Virginia Commonwealth University, 1200 E Broad Street, PO Box 980036, Richmond, VA 23298 USA

**Keywords:** Left atrial dysfunction, Heart failure, Atrial fibrillation

## Abstract

**Purpose of Review:**

To summarize the recent literature examining the degree of overlap among atrial fibrillation (AF), heart failure (HF), and left atrial (LA) dysfunction, the prognostic relevance of finding a second condition in one of the other disease states, and the treatment effects for one condition on the secondary entities.

**Recent Findings:**

If one condition (AF, HF, or LA dysfunction) is present, there are very high prevalences of a second condition demonstrated across several cohorts. Additionally, the presence of a second condition consistently has a negative impact on prognosis. Treatment effects for AF or HF tend to show bidirectional benefit, whereas data is limited toward impacts on LA dysfunction.

**Summary:**

Patients with AF, HF, or LA dysfunction often have more than one of these conditions (either clinically manifest or occult), which may influence prognosis. Established treatment pathways for AF or HF tend to show benefits in the second condition, whereas there is limited data to determine effects on LA dysfunction, or whether LA dysfunction may be directly targeted for therapy.

## Introduction

Using M-mode echocardiography it was established that LA dilation from atrial fibrillation (AF) or heart failure (HF) exhibited worse prognosis. With advancements in cardiac imaging techniques and post-processing software, more precise measurements of LA function have been established. In this review, we discuss the overlap between AF and HF, the importance of LA dysfunction which may commonly be detected through multimodality imaging in both populations, and promising future directions to advance patient care.

## Left Atrial Anatomy and Function

### Anatomy

Normally, the LA is posteriorly positioned immediately anterior to the esophagus and descending aorta [1, 2]. Several (often four) pulmonary veins enter the posterior aspect of the LA [2]. The interatrial septum divides the left and right atria, with a thin fossa ovalis representing the fusion of layers of the foramen ovale. There is significant anatomic variation in septal structure, including pouches created by layer configurations at the time of fusion, hypermobile or aneurysmal septal movement, lipomatous hypertrophy, and failure of fusion resulting in a patent foramen ovale (present in 10–35% of the population). Along the anterolateral aspect of the LA lies the LA appendage, a fingerlike projection with pectinate muscles that increase endocardial surface area (unlike the otherwise smooth endocardial surface of the body of the LA) and thus may allow it to be more sensitive to increases in LA pressure for natriuretic peptide release. The LA appendage may be found in one of several morphologies, conventionally describe as “chicken wing,” “windsock,” “cauliflower,” or “cactus” shapes, each with implications for risk of thrombosis in conditions that predispose to stasis of flow [1]. Finally, the mitral valve allows for diastolic transmission of blood from the LA to left ventricle (LV), closing during LV systole to prevent backward flow.

### Function

Normal LA function involves expanding to provide a reservoir for blood during ventricular systole, followed by the facilitation of passive movement of blood across the mitral valve into the LV during ventricular diastole (conduit function), and finally – assuming sinus rhythm – contracting during late ventricular diastole to optimally load the LV. A properly functioning LA is estimated to be responsible for approximately 15–30% of cardiac output [3]. 

**Fig. 1:**
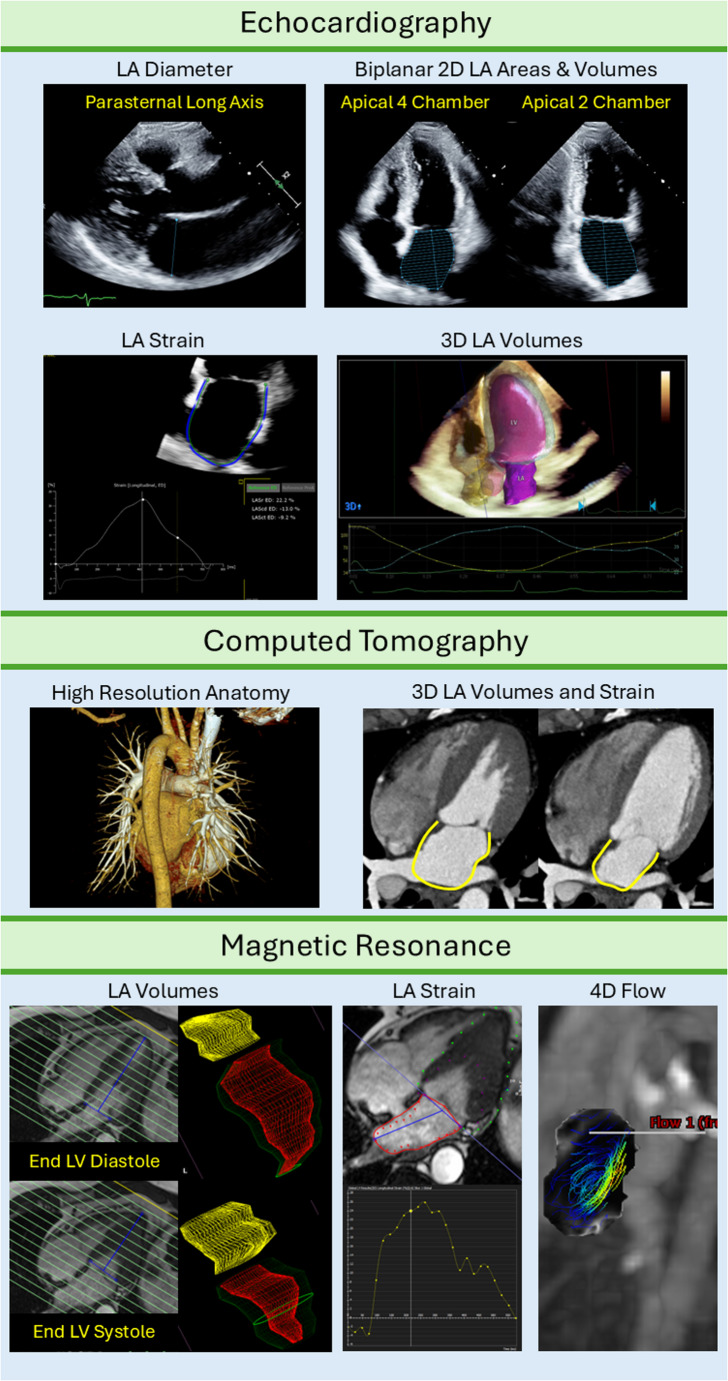
Examples of Multimodality Imaging Assessment of Left Atrial Function. Examples from multimodality imaging of LA size and function. Echocardiography allows for linear, 2D, and 3D measurements, including volumetric- and strain-based functional evaluation. Computed tomography provides high resolution anatomy visualization, also with the capacity for volumetric and strain assessments. Magnetic resonance imaging can similarly provide high resolution imaging of the LA for volumetric and strain-based characterization, as well as complex flow computations for calculating measures of stasis. LA— left atrial; LV— left ventricular

**Fig. 2:**
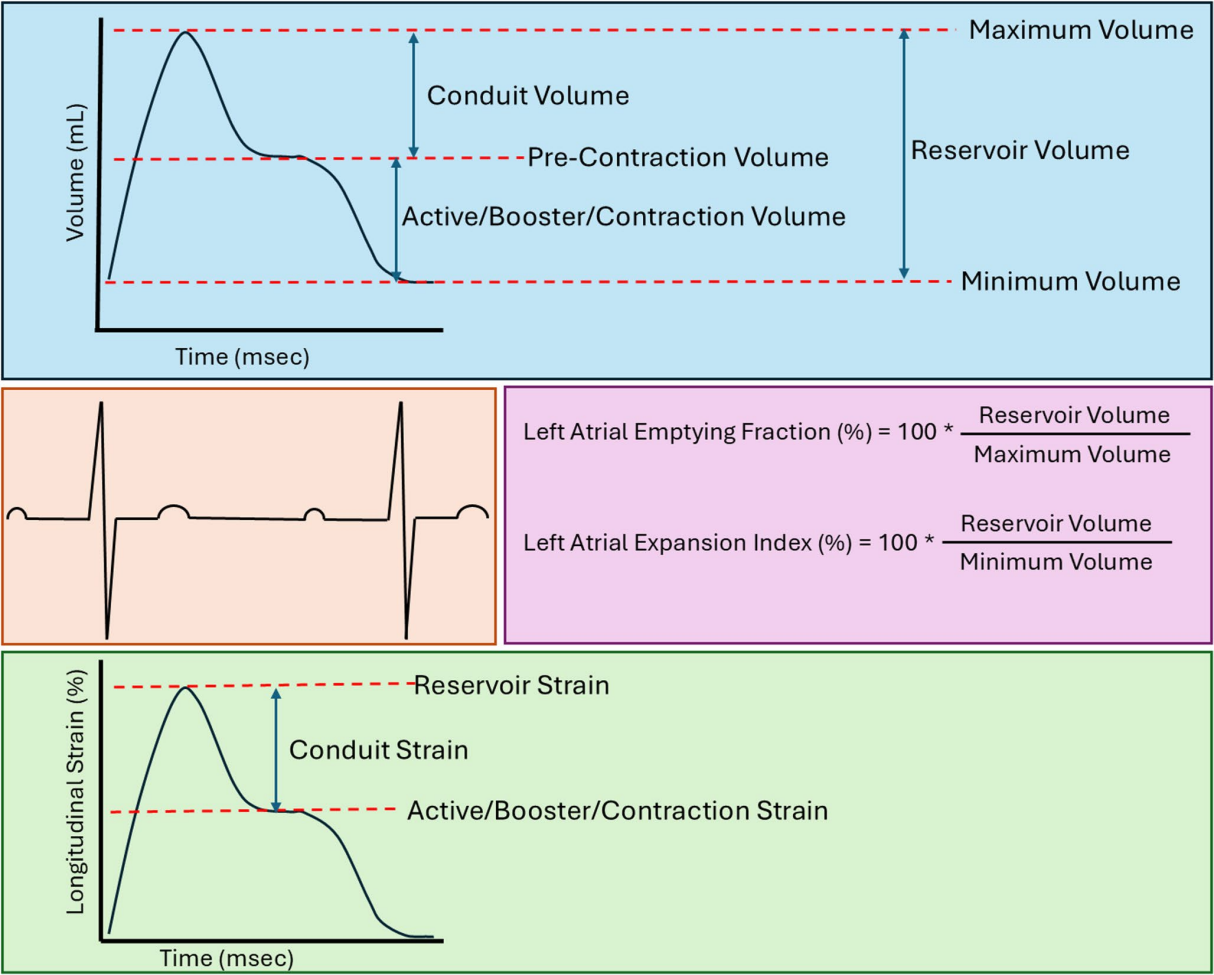
Commonly Used Measures of Left Atrial Function. Depiction of typical time-volume and time-strain curves in patients in sinus rhythm, shown relative to the electrocardiography. During ventricular systole, the LA fills and expands via the pulmonary veins. During early ventricular diastole, the mitral valve opens and allows for passive LV filling with corresponding LA decompression (conduit function). Following the P-wave, late diastole occurs which involves LA contraction, optimizing LV preload (booster function). LA— left atrial; LV— left ventricular

A variety of noninvasive techniques have been applied to measure LA size and function, each with distinct advantages and disadvantages (Table 1; Fig. 1). Transthoracic echocardiography (TTE) is widely available and can provide several metrics, albeit dependent on adequacy of acoustic windows that are typically anterior on the chest wall, furthest from the posteriorly positioned LA. Linear measurements derived from the days of M-mode echocardiography (e.g. LA diameter) have evolved to 2D measurements (e.g. LA area), and eventually 3D techniques (LA volume, either by Simpson’s biplane techniques or 3D echocardiography) to quantify maximal LA size. Current guidelines recommend volumetric techniques, ideally indexed to body surface area, as the preferred measurement that best overcomes anatomical assumptions inherent in other techniques [4]. In addition to size, LA function can be described with volumetric data, including the individual phasic components as shown in Fig. 2. While volumetric measurements obtained with 2D TTE can be susceptible to variances in slice positioning at the time of acquisition, LA strain assessment with speckle tracking allows for assessment of LA wall deformation during the cardiac cycle. Due to its ease in assessment and strong prognostic evidence, LA strain (specifically, reservoir strain) has been has been adopted recently as the primary means of defining mechanical LA myopathy by the Heart Failure Association of the European Society of Cardiology [5].

**Table 1:** Multimodality imaging of the left atrium: advantages and disadvantages

	*Transthoracic Echocardiography*	*Transesophageal Echocardiography*	*Computed Tomography*	*Cardiac Magnetic Resonance*
Advantages	• Availability • Low cost • No ionizing radiation • Several validated measures	• Proximity to left atrium, appendage, pulmonary veins	• High spatial resolution • Rapid image acquisition • Few contraindications • Not limited by acoustic windows • No geometric assumptions	• High spatial and temporal resolution • No ionizing radiation • Not limited by acoustic windows • No geometric assumptions
Disadvantages	• Limited by acoustic windows • Lower spatial resolution • Technical variability • Geometric assumptions	• Invasive • Need for sedation • Unable to visualize entire left atrium in one image	• Ionizing radiation • Limited use for functional assessment • Potential contrast reactions • Higher cost	• Claustrophobia • Higher cost • Potential contraindications • Potential contrast reactions

As compared to TTE, transesophageal echocardiography (TEE) involves positioning of the probe directly posterior to the LA, with marked improvement in visualization of several components of anatomy. Sector width is typically constrained such that full visualization of the LA is not achieved in one image (and thus functional measures are limited), but the superior visualization of the LA appendage is typically sufficient to safely exclude thrombus and allow early direct current cardioversion for patients in AF [6]. While pulmonary vein inflow and LA appendage exit velocities may be easily derived from this position (which may both be influenced by LA function), the main drawbacks of this technique include the need for sedation and relatively invasive nature.

Cardiac computed tomography (CT) is an advanced imaging option for the LA, with advantages including rapid acquisition, high spatial resolution, and the ability to perform irrespective of acoustic windows [7]. Indeed, CT may precisely identify LA appendage morphology and has shown similar performance to transesophageal echocardiography in the identification of appendage thrombus for patients in AF [8]. While volumetric or strain-based assessments are possible with CT, these come at the expense of increased exposure to ionizing radiation [9, 10]. Downsides of this technique include cost, the use of ionizing radiation, and (depending on the indication) need for iodinated contrast.

Cardiac magnetic resonance (CMR) cinematic sequences may be deployed for volumetric or strain-based measures of LA function, either in a biplanar manner or using a stack of slice offsets that advance beyond the base of the LV through the entirety of the atrial chambers. Further, flow-based assessments are possible, including with 4D techniques that can provide measures of LA stasis which can become more pronounced in conditions that promote LA dysfunction [11]. When combined with angiographic approaches, defining pulmonary vein anatomy and detecting LA appendage thrombus is possible with similar performances to other techniques [8, 11]. Limitations include possibility of patient claustrophobia, implanted ferromagnetic material which may cause image artifact and are sometimes contraindicated in the magnetic resonance environment, and (depending on the indication) need for gadolinium-based contrast.

## Overlap between Atrial Fibrillation and Heart Failure

### Atrial Fibrillation in Patients with Heart Failure

Abundant data across several cohorts have established the high prevalence of clinical diagnoses of AF in patients with HF. Indeed, AF diagnoses are often seen in at least one third of patients HF, with higher rates in patients with HF with preserved ejection fraction (HFpEF) [12–14]. Among patients with HF, a comorbid diagnosis of AF at baseline – or an incident diagnosis of AF during follow up – confers a worse prognosis [12–17], with worst outcomes in more advanced stages of AF [18]. 

In addition to clinically apparent AF, occult AF is common in patients with HF. Across several cohorts of patients with HF, ambulatory rhythm monitors have been shown to yield high rates of AF detection, ranging from 9 to 14% of patients using a 14-day patch monitors [19, 20] up to 19–26% using long term implantable recorders [21, 22]. In an ambulatory rhythm monitoring sub-study of the REDUCE LAP-HF II trial, 194 of 367 patients (53%) with HFpEF had AF detected on baseline monitors worn for 4 to 6 days, including 51 (26% of those with AF) who did not have a clinical history of AF. With continued interval rhythm monitoring throughout the next 12 months, 324 (88%) patients within the sub-study cohort had AF detected by patch monitors, including 141 patients (38% of those with AF) who did not have prior diagnoses of AF [20]. Similar to clinically apparent AF, the presence of subclinical AF is associated with worse prognosis in patients with HF [22, 23]. 

Given this high degree of overlap and impact on prognosis in patients with HF, the effects of AF treatment on clinical outcomes is of particular relevance. In a prespecified sub-analysis fo the EAST-AFNET4 trial, patients with both AF and HF experienced lower rates of a composite of death or hospitalization for HF when randomized to early rhythm control (the majority receiving medical therapy only) compared to usual care [24]. With catheter ablation, data is particularly favorable in HF with reduced ejection fraction (HFrEF). The AATAC trial randomized patients with HFrEF and cardiac implantable electronic devices to catheter ablation versus amiodarone therapy, finding fewer recurrences of AF and lower rates of death or hospitalization in those randomized to ablation [25]. Similarly, the CASTLE-AF demonstrated superiority of catheter ablation for AF in reducing mortality or HF hospitalization over medical therapy in patients with HFrEF [26]. These findings were reinforced with the CASTLE-HTx trial, which demonstrated reduced rates of death, LV assist device implantation, or heart transplantation in patients with AF and transplant-eligible HFrEF who were randomized to catheter ablation over medical therapy [27, 28]. 

The impact of AF treatment in patients with HFpEF is less well established, although several studies offer favorable signals. Early analyses suggested high overall success rates in rhythm control for patients with HFpEF who underwent catheter ablation, with improvements in echocardiographic measures of systolic and diastolic LV function in those who were able to maintain sinus rhythm [29]. Furthermore, a recent post-hoc analysis of the CABANA trial suggests that in patients meeting criteria for or with high probability of HFpEF, catheter ablation for AF is associated with a lower risk of cardiovascular death or hospitalization [30]. 

In the STALL AF-HFpEF prospective study, 20 patients with AF and comorbid HFpEF (diagnosed by exercise right heart catheterization) underwent catheter ablation and were willing to have repeat right heart catheterization after at least 6 months of follow up. Of these, 9 (45%) no longer met HFpEF criteria, notable for the fact that HFpEF regression occurred predominantly in those who did not have arrhythmia recurrence in the follow up period [31]. In a subsequent small randomized controlled trial of 31 patients, catheter ablation was again shown to be associated with HFpEF regression, improved peak oxygen consumption on exercise testing, lower natriuretic peptide biomarker levels, and higher quality of life scores [32]. This was in contrast to patients randomized to medical therapy, in whom there were no significant changes in the same measures. Again, HFpEF regression and other benefits were experienced predominantly in those who experienced arrhythmia freedom in follow up [32]. In those patients in AF at baseline testing who follow up in sinus rhythm, such improvements in exercise capacity and biomarkers is somewhat intuitive, as it has previously been shown that patients with HF with preserved or mildly reduced ejection fraction and AF have lower cardiac index at rest with lower augmentation during exercise and lower peripheral oxygen extraction compared to those in sinus rhythm [33]. 

### Heart Failure in Patients with Atrial Fibrillation

There is a similarly large body of evidence that describes the high prevalence of clinically manifest HF in patients with AF. Indeed, the H2FpEF score, a commonly used framework for diagnosing HFpEF, includes a diagnoses of AF as the highest weighted component (+ 3 points, where each increase by 1 point approximately doubles the likelihood of HFpEF diagnosis) [34]. In the Framingham cohort, 37% of patients with AF had HF diagnosed at some point in their monitored course, particularly HFpEF [13]. Those with comorbid diagnoses, including those with AF who develop a diagnosis of HF, experience worse prognosis [13, 15]. 

Occult HFpEF is common in patients with AF. In patients with AF referred for catheter ablation, patients may be found to have elevated resting LA pressures at the time of procedure in 14–15%, with an additional 19–25% of patients with elevated pressures uncovered with isometric handgrip or arm cycler ergometer exercise (total 34–39% of patients found to have occult HFpEF) [35, 36]. With exercise right heart catheterization, prevalence appears even higher. Using this technique, the STALL AF-HFpEF trial found occult HFpEF in 35 of 54 patients (65%) [31]. In patients with unexplained dyspnea referred for exercise right heart catheterization, Reddy et al. discovered criteria for HFpEF in 91% and 98% of patients with comorbid paroxysmal or persistent AF, respectively [37]. 

There is favorable data for several HF treatments in reducing AF burden. Enalapril is associated with a lower incidence of new onset AF and fewer AF-related hospitalizations in patients with HFrEF [38, 39]. Similarly, treatment with valsartan or candesartan is associated with lower incidence of AF onset in patients with HF (predominantly HFrEF cohorts) [40, 41]. In patients with HFpEF, sacubitril-valsartan did not appear to offer additional protection from AF compared to enalapril. Several trials of beta blockers have shown a reduced incidence of AF in patients with HFrEF [42, 43]. Aldosterone antagonism with eplerenone in HFrEF demonstrated reduced incidence of new AF [44], whereas post-hoc analyses of spironolactone in patients with HFpEF did not suggest AF reduction [45, 46]. Nonsteroidal aldosterone antagonism with finerenone had a strong trend toward reduced AF incidence in patients with HFpEF [47], consistent with pooled analysis from the larger investigative program incorporating patients with chronic kidney disease and diabetes mellitus [48]. 

Data is mixed to neutral regarding other HF therapies. A post-hoc analysis initially suggested reduction of new onset AF with dapagliflozin in patients with diabetes mellitus with or at high risk for atherosclerotic cardiovascular disease [49], but the same effect was not seen in a trial of dapagliflozin enrolling patients with HF [50]. A consistent lack of AF reduction was seen with empagliflozin in patients with HFpEF [51]. Data is uncertain with digoxin, although there was notably a trend toward reduced hospitalization for supraventricular arrhythmias noted in the DIG trial for patients with HFrEF treated with digoxin [52]. Despite other benefits, ivabradine has shown a consistent signal for increased risk of AF, including in patients with HFrEF [53, 54]. There was no apparent differences in new onset AF in a trial of patients with HFrEF randomized to vericiguat, and data is insufficient regarding effects on AF risk with hydralazine/isosorbide dinitrate, omecamtiv mecarbil, or glucagon-like peptide-1 receptor agonists.

## Overlap between Left Atrial Dysfunction and Heart Failure

### Left Atrial Dysfunction in Patients with Heart Failure

Signs of LA dysfunction are common in patients with HF and appear to have clinical relevance. In an observational study of patients with HF across the spectrum of LV ejection fractions, echocardiographically-derived LA reservoir strain was correlated with quality of life as measured by the Minnesota Living with Heart Failure Questionnaire [55]. In patients with HFrEF, the majority of patients have reduced LA strain (81% in one cross sectional study) [56], with LA dysfunction strongly associated with risk of downstream mortality or HF hospitalization [56, 57]. Data is consistent across CT- and CMR-derived measures of LA dysfunction [58, 59]. During acutely decompensated HF, LA strain is markedly reduced, with significant improvements after decongestion and 6 week follow up. Those patients who have persistently low LA strain after decongestion experience higher rates of death or HF readmission [60]. Similarly, LA impairments in compliance and contraction are common in HFpEF, with blunted LA functional reserve during exercise that appears linked to risk of downstream HF events [18, 61]. The impact of HF treatments on LA function is not well understood, as most studies have focused on traditional measures of LA size rather than functional analysis [62]. 

In addition to predicting adverse HF events, the presence of LA dysfunction in patients with HF may be a clue into the risk of comorbid or incident AF. Indeed, patients with HFpEF who have increasing stages of AF have worse LA function [18]. Electrocardiogram analysis has shown promise in identifying patients with lower LA strain on echocardiography, who then have higher risk of AF diagnosis in the subsequent years of follow up [63]. 

### Heart Failure Risk in Patients with Left Atrial Dysfunction

In large cohort studies, the presence of LA dysfunction appears to have relevance in predicting risk of incident HF. This may be a particularly useful warning sign, since in one cross-sectional study of healthy adults, abnormalities in LA strain seem to appear about a decade prior to traditional echocardiographic measures of diastolic dysfunction [64]. Indeed, 20% of patients from the Atherosclerosis Risk in Communities study who underwent echocardiography had LA functional abnormalities, with LA emptying fraction and strain-derived measures of phasic function associated with incident HF or death [65]. Similar findings were shown in the Copenhagen City Heart Study, where volumetric measures of LA function (emptying fraction and expansion index) were predictive of incident HF or death, albeit with only LA minimum volume maintaining predictive value on multivariable analysis [66]. In patients with undifferentiated dyspnea, the HFpEF-STRESS Trial found that exercise CMR-derived measurements of LA strain had high diagnostic performance in distinguishing HFpEF (reduced LA function) compared to noncardiac dyspnea [67]. 

## Overlap between Left Atrial Dysfunction and Atrial Fibrillation

### Left Atrial Dysfunction in Patients with Atrial Fibrillation

There is a similarly high prevalence of LA dysfunction in patients with AF. Patients with lower measures of LA function who are referred for first time catheter ablation display more extensive involvement with low voltages with endocardial mapping techniques [68]. Echocardiographic-derived LA strain worsens across advancing stages of AF and has been correlated with the presence of scar as detected by CMR late gadolinium enhanced imaging [69]. While in sinus rhythm, patients with paroxysmal AF have worse LA function compared to healthy controls, with worse still LA function in persistent AF – the degree of dysfunction again correlating to scar burden [70]. This dysfunction appears tied to the degree of stasis of flow within the LA – which is higher in patients with paroxysmal AF compared to healthy controls and worsens with established stroke risk factors [71]. Even endurance athletes with paroxysmal AF have lower LA function on echocardiography compared to well-matched athletes without AF, with better discriminatory power compared to LA volume measures and despite similar P-wave findings on electrocardiograms [72]. Patients with HFpEF appear to have stepwise decreases in LA function with increasing stages of AF compared to patients with HFpEF but no AF [18]. Treatment of AF (with cardioversion or catheter ablation) tends to favor stabilization or improvement in LA function for those who maintain rhythm control, compared to further worsening of LA function in patients with AF recurrences [73–79]. 

Again, the presence of LA dysfunction has prognostic relevance in patients with AF. Those who have reduced LA strain prior to catheter ablation have been shown in multiple cohorts to have higher rates of post-ablation AF recurrences [80–83]. Furthermore, LA emptying fraction and expansion index in patients with AF were significantly associated with risk of cardiovascular death or HF hospitalization, with better discriminatory power than LA size.

### Atrial Fibrillation Risk in Patients with Left Atrial Dysfunction

The presence of LA dysfunction has shown strong associations with risk of downstream diagnosis of AF. In the Multi-Ethnic Study of Atherosclerosis, lower LA emptying fractions were independently associated with incident AF [84]. Similar findings were seen in the Copehagen City Heart Study, even in those with normal LA sizes [85]. Patients with embolic stroke of undetermined source have high rates of occult AF, and those with reduced LA strain appear to have particularly high rates of AF detected with rhythm monitoring [86, 87]. 

## Clinical Relevance of Left Atrial Dysfunction Beyond Heart Failure and Atrial Fibrillation

### Stroke

Recent reports have demonstrated a link between LA dysfunction and stroke. Indeed, LA dysfunction (and associated stasis of flow) may play a key role in the thromboembolic mechanism associated with patients with AF. For example, the ASSERT trial found a significantly higher risk of stroke in patients who had subclinical AF as detected by with cardiac implantable electronic devices [88], but further analysis revealed that only a minority of the stroke events (4 of 26, or 15%) occurred within 30 days of an episode of AF [89]. Analyses from the Atherosclerosis Risk in Communities [90], Multi-Ethnic Study of Atherosclerosis [91], and Copenhagen City Heart Study [92] cohorts have found that reduced measures of LA function are associated with downstream risk of stroke, often independent of presence of AF or other stroke risk factors. This supports the notion that LA dysfunction can be sufficient to facilitate cardioembolic stroke, irrespective of AF [93, 94]. 

### Dementia

Cardioembolic events may occur on a smaller scale, leading to subclinical cerebral infarctions that can affect cognitive function. Data from the Cardiovascular Abnormalities and Brain Lesions study supports that LA dysfunction can be involved in this phenomenon, with greater LA volumes and reduced LA function associated with subclinical cerebrovascular disease detected by brain magnetic resonance imaging, independent of clinical risk factors including AF [95]. Echocardiographic analysis from the Atherosclerosis Risk in Communities study further supports the link between LA myopathy and incident dementia [96]. 

## Conclusion/Future Directions

Across the past several decades, bidirectional links among AF, HF, and LA dysfunction have become increasingly understood (Central Figure). Indeed, each entity is common in the other two conditions, whether clinically manifest or occult, and the presence of two or more findings together appears strongly associated with worse outcomes. Direct treatments for HF and AF have been established and continue to be refined, with encouraging signals for crossover benefits in the other conditions. What remains to be further clarified is (1) the impacts of treatment for AF or HF on LA function, (2) the role of LA functional analysis in clinical practice to trigger advanced screening for the other two conditions, and (3) whether the LA may be directly targeted for therapy to reduce the occurrences of HF, AF, and other embolic mediated clinical events.

**Figure:**
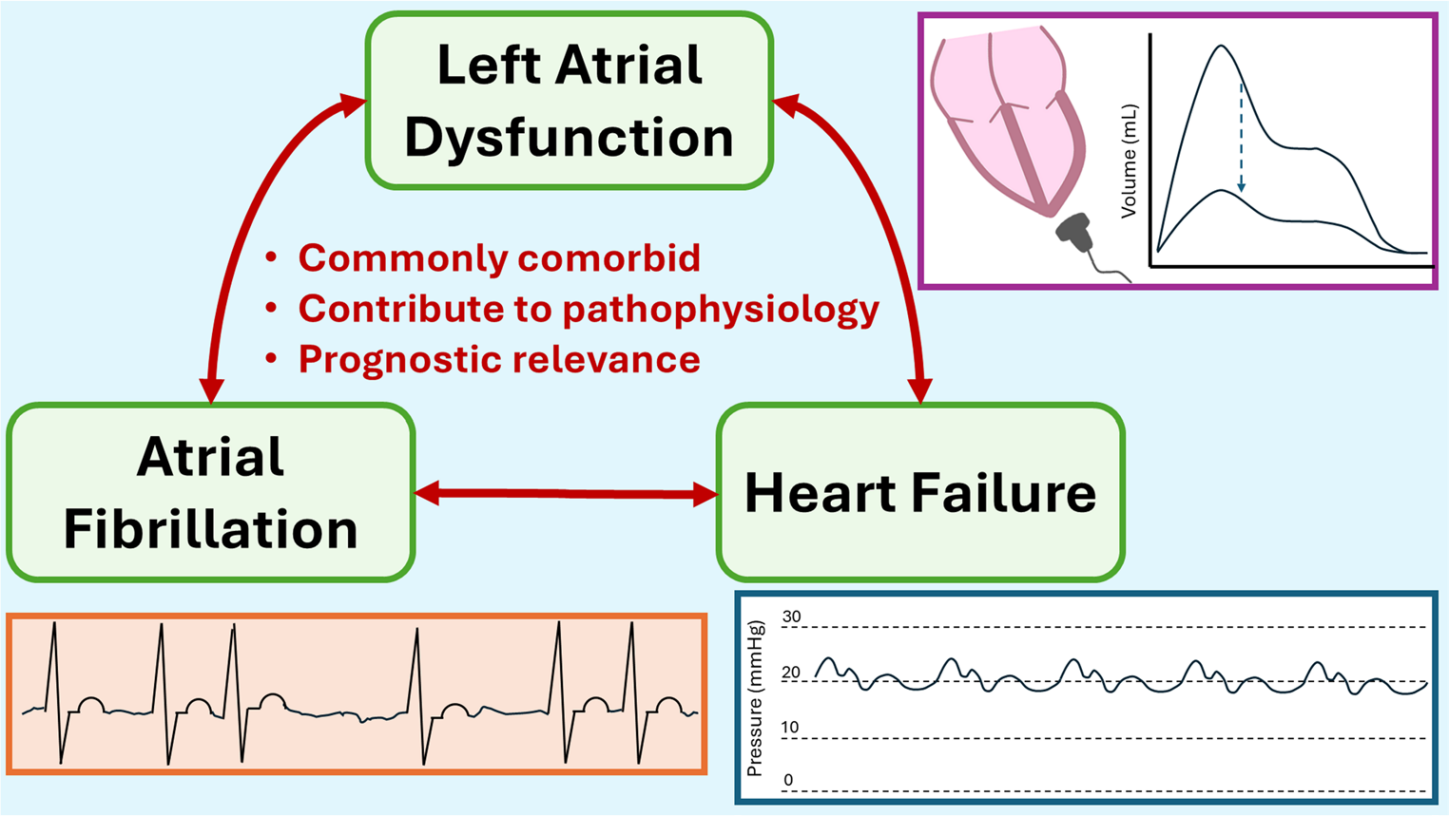
Central Figure: **Left Atrial Dysfunction in Atrial Fibrillation and Heart Failure** Due to overlapping risk factors and bidirectional causative relationships, LA dysfunction, AF, and HF commonly coexist, whether clinically manifest or occult. Having two or more overlapping conditions is associated with worse prognosis than those who only have one condition. AF—atrial fibrillation; HF—heart failure; LA— left atrial

## Key References

- Reddy YNV, Obokata M, Verbrugge FH, Lin G, Borlaug BA. Atrial Dysfunction in Patients With Heart Failure With Preserved Ejection Fraction and Atrial Fibrillation. Journal of the American College of Cardiology. 2020;76:1051–64.
  - Findings from this study indicate that in patients with heart failure with preserved ejection fraction, advancing stages of atrial fibrillation are associated with increased left atrial volumes, worsened left atrial functional parameters, higher filling pressures representing increased congestion, and worse survival.
- Inciardi RM, Claggett B, Minamisawa M, Shin SH, Selvaraj S, Gonçalves A, et al. Association of Left Atrial Structure and Function With Heart Failure in Older Adults. Journal of the American College of Cardiology. 2022;79:1549–61.
  - This analysis of older patients enrolled in the Atherosclerosis Risk In Communities study who were without prevalent heart failure, left atrial abnormalities were common and prognostic of incident heart failure or death.
- Hauser R, Nielsen AB, Skaarup KG, Lassen MCH, Duus LS, Johansen ND, et al. Left atrial strain predicts incident atrial fibrillation in the general population: the Copenhagen City Heart Study. European Heart Journal Cardiovascular Imaging. 2022;23:52–60
  - Findings from this analysis of the Copenhagen City Heart Study suggest that in healthy individuals without a diagnosis of atrial fibrillation, lower left atrial function may predict incident atrial fibrillation.

## Data Availability

No data was generated for this review article.

## Abbreviations

AF: atrial fibrillation
HF: heart failure
LA: left atrial/atrium
LV: left ventricular/ventricle
